# An Unusual Case of Kidney Injury in a Young Woman with a Connective Tissue Disease

**DOI:** 10.1155/2022/3833649

**Published:** 2022-05-30

**Authors:** Roberto Rivera, Salvador Vila

**Affiliations:** Department of Medicine, University of Puerto Rico School of Medicine, Guillermo Arbona Building Room A824A, PO Box 365067, San Juan, PR 00936-5067, USA

## Abstract

A 32-year-old female was admitted to our institution with thrombocytopenia, fever, serositis, hepatosplenomegaly, diffuse lymphadenopathy, and renal insufficiency. A diagnosis of systemic lupus erythematosus was made. Due to recalcitrant thrombocytopenia, serositis, and renal insufficiency methylprednisolone was prescribed in high doses. In addition to proteinuria and hematuria, she was found to have uric acid crystals in her urinalysis. A serum uric acid was found elevated at 18 mg/dL. Rasburicase infusions were started. Within 5 days of commencing rasburicase and continuing high-dose methylprednisolone, her serum creatinine normalized and proteinuria resolved. The microhematuria disappeared within 2 weeks of beginning rasburicase. The rapid reversal of renal insufficiency and all urinary abnormalities after the start of rasburicase infusions suggests that the renal injury was most likely due to uric acid-mediated renal injury and not lupus nephritis. Our case illustrates the co-occurrence of 2 distinct clinical entities, one common for the patient's age, sex, and foremost clinical findings, while the other uncommon and unexpected, but both associated to kidney injury. Clinicians must be aware that careful evaluation of symptoms and laboratory tests is needed to make a thorough differential diagnosis and provide the right treatment at the most opportune moment.

## 1. Case Report

A 32-year-old female was transferred to our institution due to recalcitrant thrombocytopenia and suspicion of lymphoma. Three months prior to her referral, she started to experience fever, chills, and abdominal pain. She was found to have an enlarged liver and spleen as well as lymph node enlargement in multiple body sites. An oncologist evaluated the patient and ordered diagnostic laboratory tests. However, she was hospitalized 3 days prior to return appointment with the oncologist at a different hospital due to hemoptysis and easily bruised skin. Anemia, thrombocytopenia, and renal insufficiency were noted. Blood products transfusions were given without improvement in the thrombocytopenia. She was then referred to our institution with a presumptive diagnosis of lymphoma.

**Figure 1:**
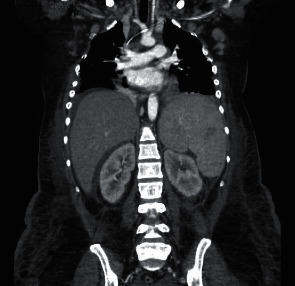
Abdominopelvic CT showing pleural effusions, lymphadenopathies, hepatosplenomegaly, and ascites.

Upon admission, a history of bouts of inflammatory arthritis in the past was obtained. Episodes of Raynaud's phenomena were observed. The CBC showed a white blood cell count of 6 930/*μ*L, hemoglobin of 7.1 gm/dL, platelet count of 3 000/*μ*L, Westergren sedimentation rate (WSR) of 150 mm/hr, and C-reactive protein (CRP) of 188.2 mg/dL. Serum chemistries were abnormal for blood urea nitrogen of 50.2 mg/dL, serum creatinine (SCr) of 2.36 mg/dL, calcium of 7.4 mg/dL, phosphorus of 4.9 mg/dL, albumin of 1.6 gm/dL, alkaline phosphatase of 246 IU/L, and serum uric acid (SUA) of 18 mg/dL. Total bilirubin and direct/indirect bilirubin ratio were normal. The urinalysis was abnormal for 35–40 RBC/HPF, and the presence of many uric acid crystals. Spot urine for protein creatinine ratio (SUPCR) was 0.815 mg/mg. Computerized tomography of the chest and abdominopelvic areas revealed bilateral pleural effusions, lymphadenopathy, hepatosplenomegaly, and ascites (Figure 1).

**Figure 2:**
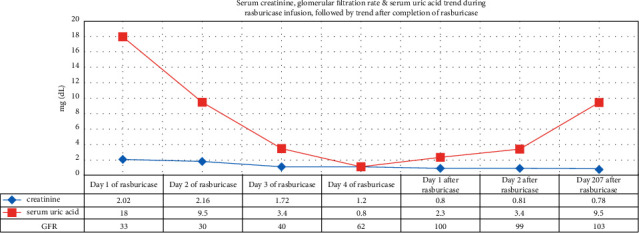
Serum creatinine, estimated glomerular filtration rate (GFR), and serum uric acid during and after rasburicase infusions.

Immediately upon arrival a clinical diagnosis of systemic lupus erythematosus with multiorgan involvement was made based on gender, age, serositis, prior history of inflammatory arthritis, renal insufficiency, and Raynaud's phenomena. Lupus nephritis was considered the cause of the kidney insufficiency and intravenous (IV) methylprednisolone, 1 gm daily for 3 days followed by 1 mg/kg/day, was started. However, the presence of uric acid crystals in the urinalysis and elevated SUA made us suspicious of urate nephropathy. Rasburicase infusions were started. Within 5 days of starting rasburicase infusions, the Scr normalized, and proteinuria resolved. Microhematuria resolved at the end of week 2 of rasburicase infusions. Urine output also increased from approximately 600 cc/day to around 1000 cc/day (Figure 2).

The platelet count failed to increase after 4 weeks of corticosteroids use and IV immune globulin (IVIG) at 0.4 gm daily for 5 days was started. One month after the IVIG infusions ended, she persisted with platelet counts below 10000/*μ*L and rituximab 750 mg IV weekly infusions for four consecutive weeks was started. Nineteen days after the start of rituximab, the platelet count began to increase, and the pleural effusions and ascites improved.

Additional investigations include a positive FANA at a titer of 1 : 2560, speckled pattern, C_3_ of 87.3 (normal range (NR) 88–201), C_4_ of 8.8 (NR 12–45), anti-SSA Ab >240 EU/mL, anti-U1 RNP Ab >206 EU/mL, RF IgM of 34 IU/mL (NR < 20),and positive lupus anticoagulant. Anti-cardiolipin, anti-*β*2 glycoprotein-1, anti-ds-DNA, anti-Sm, and anti-CCP Ab were all negative. Serum cryoglobulins were not detected. Bone marrow biopsy showed a normocellular bone marrow with megakaryocytic hyperplasia. Core needle biopsy of axillary lymphadenopathy revealed polytypic plasmacytosis with normal kappa/lambda ratios and HHV-8 negative. Flow cytometry was unremarkable and chromosomal analysis failed to reveal karyotypic abnormalities.

One year later, the patient is on prednisone 15 mg daily and hydroxychloroquine at 5 mg/Kg/day. She is not taking allopurinol. The blood counts are normal, Scr is 0.78 mg/dL, microhematuria is not present, SUPCR is 0.144 mg/mg, SUA 7.6 mg/dL, fractional excretion of uric acid (FEUA) is 6.6%, with hourly uric acid (UA) urine excretion of 18.5 mg/1.73 M^2^, WSR is 27 mm/hr, and serum complement levels are normal. During the year of follow-up, she has repeatedly tested positive for anti-ds-DNA Ab and lupus anticoagulant and has developed synovitis of hand joints. A positron emission tomography showed F-18 FDG avidity in lymph nodes of the cervical, axillary, retroperitoneal, and pelvic areas. Excisional cervical lymph node biopsy was conducted, and a polytypic lymphoplasmacytic infiltrate was seen. Neither storiform fibrosis nor obliterative phlebitis was observed. Markers for lymphoproliferative malignancies remained negative. She has not required further rituximab infusions.

## 2. Discussion

Autoimmune rheumatic diseases may start as illnesses that involve multiple body organs from its outset [1, 2]. In such cases, organ damage is usually considered to be due to the autoimmune disease. Our patient meets criteria for a definitive diagnosis of systemic lupus erythematosus (SLE) [3, 4]. The prevalence of SLE in the Hispanic population in the US has been reported around 84.6/100 000 [5]. The prevalence of SLE in Puerto Ricans has been reported to be higher with an overall prevalence of 159/100 000, and of 277/100 000 in females [6]. Kidney injury is a frequent manifestation of SLE with a prevalence of lupus nephritis (LN) upward of 50% [7]. LN has to be aggressively treated to prevent irreversible loss of renal function, chronic renal insufficiency, and the potential need for renal replacement therapy.

Other potential causes of kidney disease in a patient with a lymphoplasmacytic infiltrate in a lymph node biopsy and an inflammatory process include multicentric Castleman disease (CD) and IgG4-related disease. Since she is HHV-8- and HIV-Ab-negative, idiopathic Castleman disease (ICD) and its variant thrombocytopenia, anasarca, retroperitoneal fibrosis, renal dysfunction, and organomegaly (TAFRO) syndrome have to be considered. ICD and TAFRO have a strong inflammatory process with systemic symptoms such as fever, elevation of the CRP and WSR, organomegaly, lymphadenopathies in multiple body sites, and cytopenia. However, international diagnostic criteria state that in order to make a diagnosis of ICD/TAFRO, inflammatory diseases such as SLE must be ruled out [8]. More recently, case reports have been published where an initial diagnosis of definitive SLE has been made, only to be changed to ICD/TAFRO over time [9, 10]. The authors described SLE as a mimicker for ICD/TAFRO. In our patient, long-term observation validated the diagnosis of SLE since over time, she developed arthritis, documented by a rheumatologist, the anti-ds-DNA Ab became positive, and the ANA positivity persisted. IgG4-related disease (igg4-rd) is highly unlikely despite the presence of lymph node biopsy showing lymphoplasmacytic infiltrates. Absence of storiform fibrosis and obliterative phlebitis in the lymph node biopsy does not rule out igg4-rd as these pathological findings are not seen in lymph nodes. However, igg4-rd is usually a disease of males in their middle age, clinical presentation and inflammatory markers tend to be more indolent, and she lacks typical renal radiographic CT changes such as enlarged kidneys and multiple nodules. In addition, patients with igg4-rd and renal disease not infrequently have retroperitoneal, pancreas, thyroid, or other organ involvement all absent in this patient [11, 12].

Uric acid nephropathy is suggested by SUA above 15 mg/dL, presence of uric acid crystals in the urine sediment, renal insufficiency, and ratio of urine uric acid to creatinine above 1.0 [12]. Hyperuricemia is believed to injure the kidney through several mechanisms including uric acid intrarenal stone formation and obstruction, tubular cell inflammation and apoptosis, decreased renal circulation, and increased oxidative stress [13]. In our patient, uric acid nephropathy is suggested by a SUA above 15 mg/dL, presence of uric acid crystals in the urine, renal insufficiency that reverted back to a normal SCr within 5 days of starting rasburicase together with an increase in urine output, and quick resolution of the microhematuria. A renal biopsy to document the presence of LN was initially not performed due to the protracted thrombocytopenia. Later, in the patient's clinical course, it was not considered due to normal SCr levels and urine sediment and the fact the authors have never seen a patient with LN with moderate renal insufficiency achieving a full renal remission within 14 days of starting corticosteroid therapy. A renal biopsy to make a tissue diagnosis of urate nephropathy was considered. However, we decided against recommending the biopsy taking in consideration the patient's risk vs benefit ratio. It was possible the kidney biopsy would not show urate acid crystals because by the time the platelet count stabilized and the biopsy could have been conducted in a safer manner, she had already finished all rasburicase infusions and was on allopurinol. In addition, even if the biopsy had shown urate deposits in the renal tubules, the probability of a change in therapy was considered remote.

The cause of the SUA elevation at admission is not clear. Conditions associated to an abrupt rise in SUA such as tumor lysis syndrome [14], rhabdomyolysis [15], and repeated epileptic seizures [16] were absent. Conditions associated to a gradual increase in the SUA were also not identified [16–19]. A genetic cause for serum uric acid elevation is highly unlikely as she has a large family with many first and second degree relatives and a negative family history of gout or uric acid stones. One year after her discharge she continues to have a slight elevation of the SUA off allopurinol. The FEUA is normal suggesting the SUA elevation is not caused by an overproduction of uric acid. It is possible she may have experienced an increase in the SUA level several weeks prior to her admission presumably due to active tissue necrosis resulting in increased uric acid renal clearance and uric acid nephropathy.

In summary, we report a case of a woman with kidney insufficiency in whom 2 different clinical entities that cause kidney injury co-occurred. SLE, a not uncommon disease in females in their 4^th^ decade of life that causes kidney injury through autoimmune mechanisms, and urate nephropathy, an extremely rare cause of kidney injury, were seen in a young female in the absence of an identifiable cause for severe hyperuricemia, that causes kidney injury through uric acid deposition in renal tissues and immune-mediated mechanisms. Methylprednisolone infusions alone, prescribed specifically for LN, would have most likely controlled the urate nephropathy through its anti-inflammatory effects since there was no evidence of uric acid stones physically obstructing urinary flow. In this respect, steroids alone would have treated two different pathogenic causes of renal injury that share similar mechanisms of injury. However, the combination of methylprednisolone and rasburicase infusions most likely accelerated the control of urate nephropathy reducing the possibility of long-term renal damage [20, 21].

Clinicians must not forget that performing a differential diagnosis is necessary in arriving at a correct diagnosis and to provide opportune treatment, in order to prevent unnecessary morbidity, particularly, since it is not always the case that different clinical entities respond to the same treatment.

## Data Availability

The clinical and laboratory data used to support the findings of this study are included within the article.
